# Rapid Development of Bleeding Esophageal Varices after Placement of Continuous Flow Left Ventricular Assist Device

**DOI:** 10.1155/2019/8410420

**Published:** 2019-11-06

**Authors:** Kaushal Majmudar, Michael Northcutt, Robert Gordon, Claus J. Fimmel

**Affiliations:** ^1^Department of Medicine, Division of Internal Medicine, NorthShore University HealthSystem, Evanston, IL, USA; ^2^Department of Medicine, Division of Gastroenterology, NorthShore University HealthSystem, Evanston, IL, USA; ^3^Department of Medicine, Division of Cardiology, NorthShore University HealthSystem, Evanston, IL, USA

## Abstract

We describe a patient with compensated cirrhosis and portal hypertension who underwent continuous flow LVAD implantation. Shortly after LVAD implantation, the patient developed new onset bleeding esophageal varices and ultimately had a fatal outcome. Our experience suggests that even well-compensated cirrhotic patients with significant portal hypertension are at risk of variceal bleeding after LVAD placement.

## 1. Introduction

Upper gastrointestinal bleeding—typically due to arterio-venous malformations—is common in patients with continuous flow left ventricular assist device (CF-LVAD), and is due to acquired von Willebrand disease and systemic anticoagulation [1–3]. We present a patient with compensated cirrhosis in whom large esophageal varices developed within a few weeks of LVAD placement, resulting in hemorrhage and eventual fatal outcome.

## 2. Case Presentation

A 62-year old male with a history of nonischemic cardiomyopathy presented with recurrent pulmonary edema requiring intra-aortic balloon pump (IABP) to maintain stability. On admission, EF was 19% and cardiac catheterization demonstrated no coronary artery disease. Given his recurrent pulmonary edema, a workup for CF-LVAD placement was initiated.

**Figure 1:**
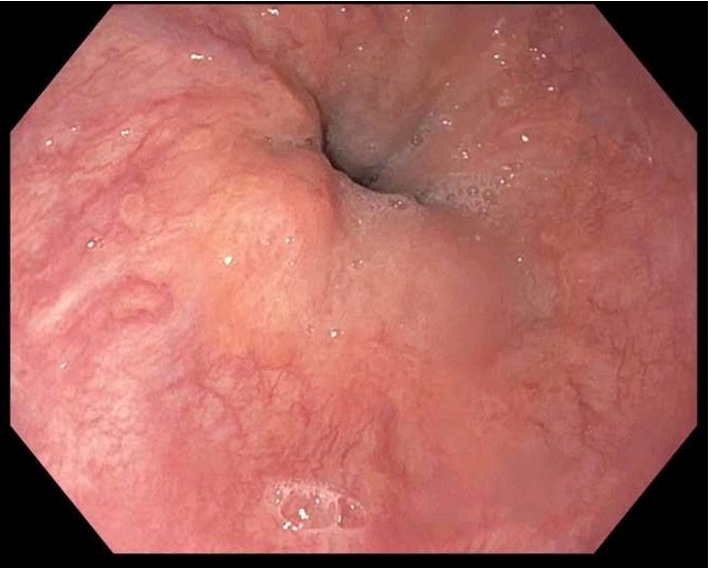
Normal gastroesophageal junction prior to CF-LVAD placement. No evidence of esophageal varices 4 days prior to LVAD placement.

**Figure 2:**
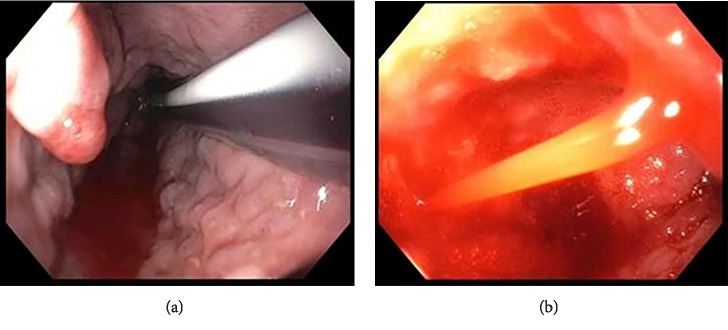
New onset esophageal varices 17 days after CF-LVAD placement. (a) Red wale sign with multiple esophageal varices in distal esophagus. (b) Active bleeding from new onset esophageal varices.

An abdominal CT scan unexpectedly revealed cirrhotic liver changes. The patient had no physical exam stigmata of advanced liver disease. Albumin, INR, transaminases, ferritin, and bilirubin were normal, and the platelet count was 170,000. Viral hepatitis serologies were negative. Upper GI endoscopy revealed no esophageal varices (Figure 1). A transjugular liver biopsy performed with the patient on IABP support, revealed a right atrial pressure of 0 mmHg and hepatic vein wedge pressure of 21 mmHg, with a portal-systemic gradient of 20 mmHg. The liver histology confirmed cirrhosis without specific etiology. The patient's Child-Pugh class A was consistent with compensated cirrhosis. He underwent uneventful placement of a HeartWare™ HVAD™ system (HeartWare, Framingham, MA). The patient was bridged from heparin to warfarin and started on aspirin. Seventeen days after LVAD placement, the patient sustained an acute upper GI bleeding episode with a drop of the hemoglobin from 8.6 to 4.9 g/dL. At the time, his INR was 2.4, and the platelet count 188,000. Emergent EGD revealed large, actively bleeding esophageal varices (Figure 2). Esophageal variceal band ligation was performed and hemostasis was achieved. An echocardiogram showed normal right ventricular function with no signs of right heart dilation or right heart failure. A Doppler abdominal ultrasound demonstrated patient hepatic veins with no evidence of Budd–Chiari. Over the following days, the patient developed ascites, hepatic encephalopathy and renal failure.

Twelve days later, the patient developed recurrent GI bleeding. EGD revealed active bleeding in the esophagus and a large clot. No specific bleeding source could be identified, and endoscopic attempts at hemostasis were unsuccessful. Placement of a transjugular, intrahepatic portosystemic shunt was considered, but the patient's MELD score of 40 was prohibitively high. The patient continued to bleed despite maximal medical support, and he expired on the following morning.

## 3. Discussion

We hypothesize that the fluid shifts and increased hepatic arterial inflow following LVAD placement caused rapid increases in esophageal variceal pressures and variceal bleeding in our patient. His low cardiac output prior to LVAD placement, likely masked the extent of his portal hypertension. In hindsight, the markedly elevated pre-LVAD transhepatic venous pressure gradient was the clearest predictor of post-LVAD variceal complications in this patient. In addition, his moderately elevated MELD score of 11 prior to CF-LVAD placement suggested a moderately elevated risk for hepatic decompensation, despite a favorable Child-Pugh score [4].

We considered the possibility that the patient's portal hypertension became more severe as a result of right heart failure and acute congestive hepatopathy after CF-LVAD implantation. This is a relatively common complication that occurs in 28% of CF-LVAD patients a median of 14 days after placement [5]. However, the normal echocardiogram on the day of his first bleeding episode argues against this possibility.

Considering the significant resource requirements and medical risks of LVAD programs, judicious patient selection is critical. No published LVAD guidelines explicitly rule out the selection of all patients with cirrhosis [6]. Our experience suggests that even compensated cirrhotic patients with significant portal hypertension are at risk for variceal bleeding after LVAD placement.

## 4. Conclusion

Patients with compensated cirrhosis and significant portal hypertension are at risk for variceal bleeding after continuous flow LVAD placement. Pre-LVAD transhepatic venous pressure gradient may be a predictor of post-LVAD complications in a patient with compensated cirrhosis.
